# Comprehensive Management of HIV With Multiple Opportunistic Infections: A Case Report

**DOI:** 10.1002/ccr3.71216

**Published:** 2025-10-08

**Authors:** Mohammad Javad Boozhmehrani, Akbar Hoseinnejad, Sara Lesani, Sara Afzalzadeh, Aryan YousefiFard, Ali Rezaei‐Matehkolaei, Sharif Maraghi, Mehdi Tavalla, Mojtaba Taghizadeh Armaki, Jalal Jafarzadeh, Mohammad‐Navid Bastani

**Affiliations:** ^1^ Department of Medical Parasitology, School of Medicine Ahvaz Jundishapur University of Medical Sciences Ahvaz Iran; ^2^ Student Research Committee Ahvaz Jundishapur University of Medical Sciences Ahvaz Iran; ^3^ Department of Medical Mycology, School of Medicine Ahvaz Jundishapur University of Medical Sciences Ahvaz Iran; ^4^ Department of Parasitology and Mycology, School of Medicine Isfahan University of Medical Sciences Isfahan Iran; ^5^ Department of Infectious and Tropical Diseases, Razi Hospital, Faculty of Medicine Ahvaz Jundishapur University of Medical Sciences Ahvaz Iran; ^6^ Infectious and Tropical Diseases Research Center, Health Research Institute Ahvaz Jundishapur University of Medical Sciences Ahvaz Iran; ^7^ Department of Pharmaceutics, School of Pharmacy Ahvaz Jundishapur University of Medical Sciences Ahvaz Iran; ^8^ Iran Zamin Medical Diagnostic Laboratory Naderi Grand Medical Complex Ahvaz Iran; ^9^ Department of Parasitology and Mycology, School of Medicine Islamic Azad University Ahvaz Iran; ^10^ Infectious Diseases and Tropical Medicine Research Center, Health Research Institute Babol University of Medical Sciences Babol Iran; ^11^ Virology Department, School of Medicine Ahvaz Jundishapur University of Medical Sciences Ahvaz Iran

**Keywords:** candidiasis, cryptosporidiosis, HIV, opportunistic infections, tuberculosis

## Abstract

Effective management of human immunodeficiency virus patients with multiple opportunistic infections requires a multidisciplinary approach, timely initiation of antiretroviral therapy, and careful adjustment for drug–drug interactions. Coordinated care and vigilant monitoring significantly improve survival and highlight the importance of individualized treatment strategies in complex co‐infection scenarios.

## 1 Introduction

The human immunodeficiency virus (HIV) continues to affect millions worldwide, with an estimated 37 million people living with HIV (PLWH) by the end of 2015. Despite a plateau in new infections at around 2.5 million annually, the burden of co‐infections in PLWH remains substantial [1]. Studies show that 10%–28% of PLWH are co‐infected with hepatitis B virus (HBV) [2]. Opportunistic infections further complicate management: cryptosporidiosis has an overall pooled prevalence of 14.5% (95% CI 10.4–19.9) among AIDS patients [3], tuberculosis prevalence ranges from 3% to 47.9% across countries [4], and candidiasis is common, with recent studies reporting point prevalence rates of 25%–50% [5, 6].

The concurrent treatment of these infections presents major challenges due to drug interactions and the need for careful therapeutic sequencing. While protocols exist for individual infections, little guidance is available for managing multiple infections in advanced HIV disease. This report describes a structured management approach in a critically ill HIV patient with multiple co‐infections, highlighting the importance of timely intervention and meticulous drug interaction management.

## 2 Case History/Examination

A 37‐year‐old married man in critical condition, presenting with diarrhea, vomiting, fever, orthostatic hypotension, anorexia, productive cough, and a diffuse throbbing headache for 8 days, was admitted to the ICU at Razi Hospital in Ahvaz, Iran, on October 25, 2022. He was diagnosed with HIV in 2013 but discontinued antiretroviral therapy a year before admission. He had a history of heroin addiction managed with methadone, which he abruptly discontinued. His social history included a 20‐pack‐year smoking history. Physical examination revealed cachexia, dehydration, and respiratory distress.

Laboratory evaluation revealed multiple significant abnormalities. The patient demonstrated severe immunodeficiency with a CD4 count of 70 cells/μL (7% of total lymphocytes). Complete blood count showed pancytopenia: white blood cell count was 3200/μL with a differential of 78% neutrophils, 15% lymphocytes, 5% monocytes, and 2% eosinophils. Red blood cell count was 2.56 M/μL with hemoglobin of 6.1 g/dL, and peripheral blood smear revealed microangiopathic changes including anisocytosis, hypochromia, schistocytes, and teardrop cells. Platelet count was markedly elevated at 830,000/μL. Inflammatory markers were significantly elevated: LDH 2398 U/L, D‐dimer 2530 ng/mL, and fibrinogen > 1200 mg/dL, suggesting a hypercoagulable state.

Microbiological studies yielded multiple positive results: stool examination confirmed *Cryptosporidium parvum* infection, bronchoalveolar lavage (BAL) revealed probable invasive pulmonary candidiasis due to *Candida albicans* (with susceptibility testing showing sensitivity to fluconazole), and sputum analysis confirmed pulmonary tuberculosis. Drug susceptibility testing of *Mycobacterium tuberculosis* showed sensitivity to all first‐line anti‐tuberculosis medications.

Viral serology demonstrated positive HBV markers, and HBV molecular testing was also positive, confirming chronic infection. Serological testing for hepatitis C virus (HCV) was positive, but molecular PCR testing was negative, indicating no active HCV replication. Given the initial presentation with fever, hypotension, and respiratory distress, sepsis was the leading differential diagnosis; however, repeated blood cultures were negative, and PCR testing also excluded COVID‐19 and influenza, thereby supporting the final diagnosis of multiple opportunistic infections in the context of advanced HIV.

## 3 Methods/Investigations and Treatment

**FIGURE 1:**
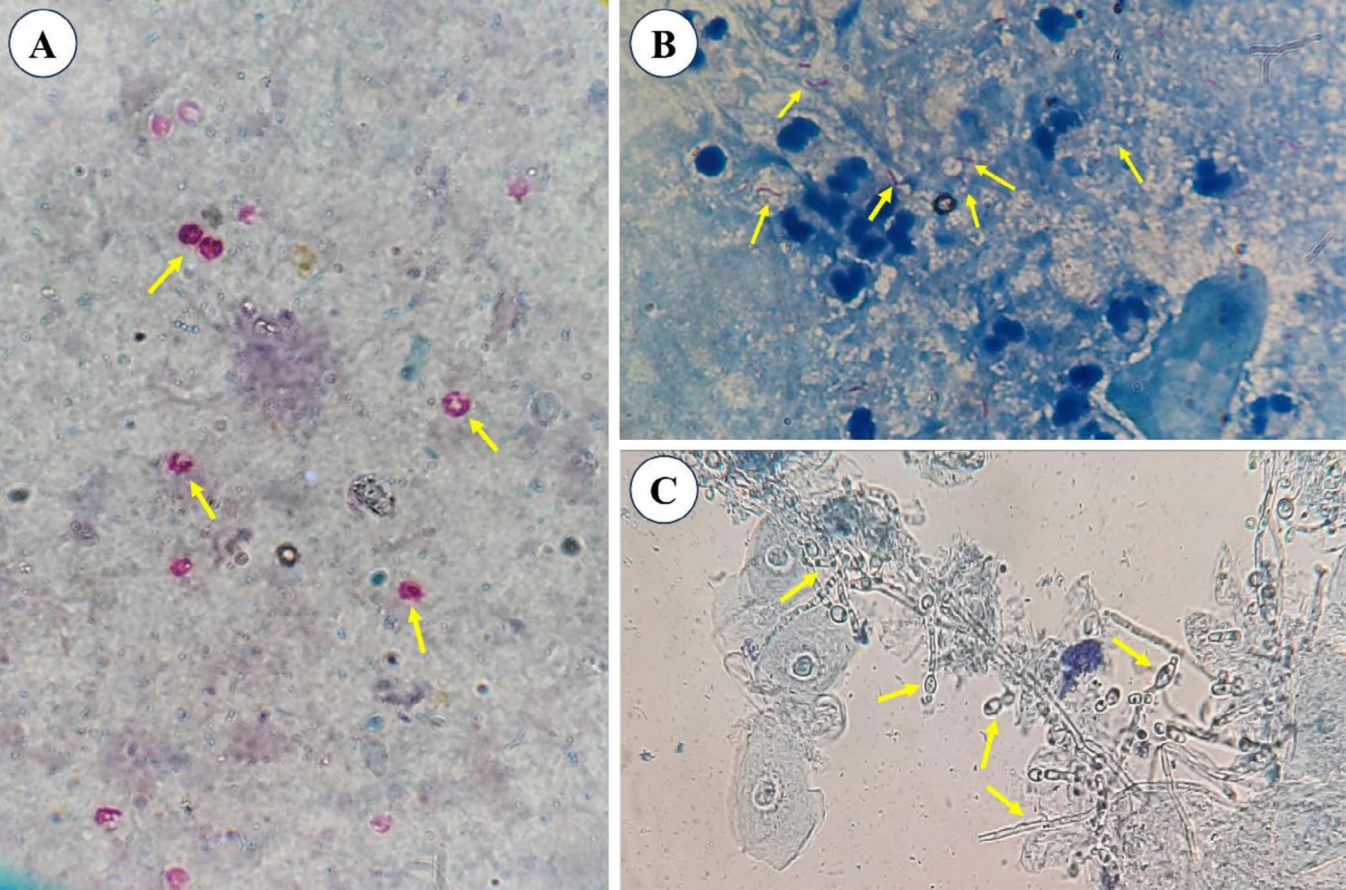
Microscopic findings of stool and bronchoalveolar lavage samples. (A) *Cryptosporidium* oocysts (yellow arrows) identified in a stool sample at 1000× magnification. (B) *Mycobacterium tuberculosis* bacilli (yellow arrows) identified in a bronchoalveolar lavage sample at 1000× magnification. (C) Pseudohyphae and *Candida albicans* yeast (yellow arrows) identified in a bronchoalveolar lavage sample at 400× magnification.

**FIGURE 2:**
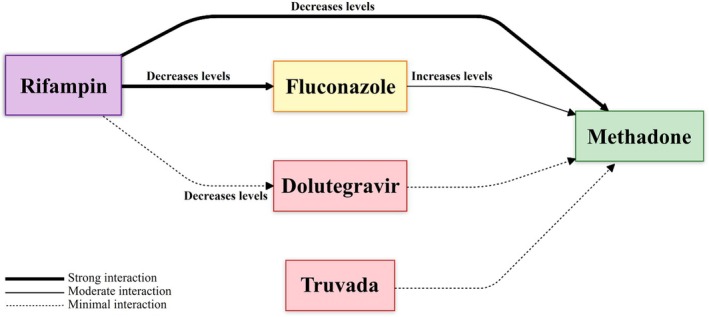
Key drug interactions in the management of multiple co‐infections.

Treatment was initiated in a carefully planned sequence, beginning with emergency stabilization on Day 1 using IV fluids and empiric broad‐spectrum antibiotics (meropenem and vancomycin) due to the patient's critical condition and suspected sepsis. Methadone was immediately reinstated to prevent withdrawal complications. Concurrently, antiretroviral therapy (dolutegravir plus Truvada) and anti‐tuberculosis treatment (HRZE regimen) were initiated to address severe immunosuppression, as indicated by a CD4 count of less than 100 cells/μL. Truvada, with its dual activity against both HIV and HBV, was included in the regimen. Routine liver examinations were conducted to monitor for hepatomegaly, a known side effect of this medication. By Day 3, fluconazole was added to manage pulmonary candidiasis after confirming adequate renal function and tolerance to the anti‐TB medications. This aggressive treatment approach, while carrying potential risks of immune reconstitution inflammatory syndrome (IRIS), was deemed necessary given the patient's critical condition and profound immunosuppression. Cryptosporidiosis was managed through immune restoration rather than specific anti‐parasitic therapy, as the latter has limited efficacy in cases of severe immunosuppression. Key drug interactions were meticulously managed to optimize therapeutic outcomes (Figure 1). The dolutegravir dose was doubled to counteract rifampin‐induced enzyme induction, while fluconazole dosing was adjusted based on renal function and its interaction with rifampin. Methadone dosage was carefully titrated to account for the combined effects of rifampin and fluconazole (Figure 2). Detailed dosing regimens and adjustments are outlined in Table 1.

**TABLE 1:** Medication dosing regimen and timing.

Medication category	Drug	Initial dose	Adjusted dose	Timing	Duration	Adjustment rationale
Antiretroviral	Dolutegravir	50 mg daily	50 mg twice daily	Day 1	Ongoing	Rifampin interaction
Antiretroviral	Truvada (Emtricitabine/Tenofovir)	200/300 mg daily	No adjustment	Day 1	Ongoing	—
Anti‐TB	Rifampin	600 mg daily	No adjustment	Day 1	6 months	—
Anti‐TB	Isoniazid	300 mg daily	No adjustment	Day 1	6 months	—
Anti‐TB	Pyrazinamide	25 mg/kg/day	No adjustment	Day 1	2 months	—
Anti‐TB	Ethambutol	20 mg/kg/day	No adjustment	Day 1	2 months	—
Antifungal	Fluconazole	200 mg daily	300 mg daily	Day 3	14 days	Renal function and rifampin interaction
Addiction management	Methadone	20 mg daily	50 mg daily	Day 1	Ongoing	Balanced between rifampin induction and fluconazole inhibition

## 4 Results/Outcome and Follow‐Up

After 6 weeks of treatment, the patient demonstrated significant clinical improvement, with resolution of initial symptoms and an increase in CD4 count to 150 cells/μL, reflecting the effectiveness of the tailored therapeutic approach.

## 5 Discussion

The concurrent management of multiple opportunistic infections in HIV requires an integrated approach. This patient's condition was complicated by severe immunosuppression, leading to co‐infections with TB, HBV, candidiasis, and cryptosporidiosis. The following key elements were crucial in achieving successful outcomes:

### 5.1 Co‐Infection Complexity

HIV compromises the immune system, making individuals susceptible to various infections. TB is one of the most common opportunistic infections in HIV patients and requires prompt initiation of anti‐TB therapy [7]. Cryptosporidiosis and candidiasis further complicate management, as both are indicative of profound immunosuppression (CD4 < 100 cells/μL) [8, 9]. Cryptosporidiosis, unlike other infections, lacks highly effective antimicrobial therapy, emphasizing the importance of immune restoration [10].

### 5.2 Drug Interactions and Adjustments

The interplay between ART, anti‐TB therapy, and antifungal agents required careful dose modifications. Rifampin is a potent CYP450 inducer, reducing the efficacy of dolutegravir, necessitating a dose increase to 50 mg twice daily [11]. Additionally, rifampin accelerates methadone metabolism, increasing the risk of opioid withdrawal, which required gradual methadone dose titration [12]. Fluconazole's inhibitory effect on CYP3A4 countered rifampin's induction, necessitating close monitoring for methadone toxicity [13].

### 5.3 Timing of ART Initiation

WHO guidelines suggest initiating ART within 2 weeks of TB treatment in severely immunocompromised patients (CD4 < 50 cells/μL) to reduce mortality, despite the risk of IRIS [14, 15]. Given this patient's critical state, ART and anti‐TB therapy were started simultaneously, with close monitoring for IRIS manifestations such as paradoxical worsening of TB symptoms.

### 5.4 Role of Supportive Care

Managing dehydration and electrolyte imbalances was essential due to severe diarrhea caused by cryptosporidiosis. The patient required IV fluids and electrolyte replacement to maintain hemodynamic stability. Nutritional support played a key role in improving overall prognosis, ensuring adequate caloric intake to counteract the effects of cachexia and malabsorption.

### 5.5 Clinical Outcome and Monitoring

After 6 weeks, the patient's CD4 count improved to 150 cells/μL, demonstrating the effectiveness of the treatment strategy. ART adherence remains crucial for long‐term success, requiring ongoing counseling to prevent discontinuation and relapse of opportunistic infections. Follow‐up evaluations were scheduled to monitor liver function, ART efficacy, and TB resolution.

### 5.6 Comparison With Reported Cases

Similar cases of advanced HIV/AIDS complicated by multiple opportunistic infections have been described in the literature. Reports include critically ill patients with concurrent infections such as cytomegalovirus, candidiasis, cryptococcosis, histoplasmosis, *Pneumocystis jirovecii* pneumonia, and *Mycobacterium avium* complex [16, 17]. These studies emphasize the diagnostic challenges, the complexity of therapeutic decisions, and the high mortality associated with such presentations. Our case shares similarities with these reports in terms of diagnostic complexity and the need for integrated management of multiple pathogens, but it differs in the specific combination of opportunistic infections and in achieving a favorable short‐term clinical outcome following a carefully sequenced treatment strategy.

## 6 Conclusion

This case underscores the importance of an integrated, multidisciplinary approach in managing HIV with multiple opportunistic infections. Careful drug interaction monitoring, timely ART initiation, and individualized treatment strategies contribute to positive patient outcomes.

## Author Contributions

**Mohammad Javad Boozhmehrani:** conceptualization, methodology, project administration, visualization, writing – original draft, writing – review and editing. **Akbar Hoseinnejad:** methodology, supervision, writing – review and editing. **Sara Lesani:** data curation, visualization, writing – original draft. **Sara Afzalzadeh:** project administration, supervision, writing – review and editing. **Aryan YousefiFard:** investigation, methodology, resources. **Ali Rezaei‐Matehkolaei:** investigation, methodology, writing – review and editing. **Sharif Maraghi:** methodology, project administration, writing – review and editing. **Mehdi Tavalla:** conceptualization, project administration, supervision, writing – review and editing. **Mojtaba Taghizadeh Armaki:** investigation, methodology. **Jalal Jafarzadeh:** investigation, methodology. **Mohammad‐Navid Bastani:** investigation, methodology.

## Disclosure

Transparency statement: The authors affirm that this research is an accurate, transparent, and honest account of the study being reported. No important aspects of the research have been omitted, and any discrepancies from the study as planned have been explained.

## Ethics Statement

The study protocols were reviewed and approved by the Ethics Committee of Ahvaz Jundishapur University of Medical Sciences, Ahvaz, Iran (Approval ID: IR.AJUMS.REC.1401.596).

## Consent

Written informed consent was obtained from the patient for publication of this case in both print and electronic formats, including images and clinical details, in Clinical Case Reports journal. The consent covers use in all formats including print, electronic, website versions, translations, licensed reprints, and derivative works. The signed consent form is retained by the authors in accordance with institutional policy and journal requirements and can be made available to the journal upon reasonable request. The patient agreed that their clinical information and images could be used for educational and research purposes while maintaining the confidentiality of personally identifying information.

## Conflicts of Interest

The authors declare no conflicts of interest.

## Data Availability

The data that support the findings of this research are available from the corresponding author, Mehdi Tavalla, upon reasonable request.
